# A Digital Image Correlation Technique for Laboratory Structural Tests and Applications: A Systematic Literature Review

**DOI:** 10.3390/s23239362

**Published:** 2023-11-23

**Authors:** Mohammed Abbas Mousa, Mustafasanie M. Yussof, Thulfiqar S. Hussein, Lateef N. Assi, SeyedAli Ghahari

**Affiliations:** 1 Department of Roads and Transportation, University of Al-Qadisiyah, Al Diwaniyah 58002, Iraq; thulfiqar.hussein@qu.edu.iq; 2 Department of Civil Engineering, Engineering Campus, Universiti Sains Malaysia, Nibong Tebal 14300, Malaysia; cemustafa@usm.my; 3 Faculty of Civil Engineering, Universiti Teknologi Malaysia (UTM), Skudai 81310, Malaysia; 4 Terracon Consultants Inc., 9522 E 47th Pl, Tulsa, OK 74145, USA; lateef.assi@terracon.com; 5 Department of Civil Engineering, Purdue University, West Lafayette, IN 47907, USA; sghahari@purdue.edu

**Keywords:** digital image correlation, surface displacements, structural monitoring, full-scale test design

## Abstract

Digital image correlation (DIC) is an optical technique used to measure surface displacements and strains in materials and structures. This technique has demonstrated significant utility in structural examination and monitoring. This manuscript offers a comprehensive review of the contemporary research and applications that have leveraged the DIC technique in laboratory-based structural tests. The reviewed works encompass a broad spectrum of structural components, such as concrete beams, columns, pillars, masonry walls, infills, composite materials, structural joints, steel beams, slabs, and other structural elements. These investigations have underscored the efficacy of DIC as a metrological instrument for the precise quantification of surface deformation and strain in these structural components. Moreover, the constraints of the DIC technique have been highlighted, especially in scenarios involving extensive or complex test configurations. Notwithstanding these constraints, the effectiveness of the DIC methodology has been validated as a strain measurement instrument, offering numerous benefits such as non-invasive operation, full-field measurement capability, high precision, real-time surveillance, and compatibility with integration into other measurement instruments and methodologies.

## 1. Introduction

In recent years, the evaluation and monitoring of material behavior and structural performance have become critical aspects of civil engineering research. Laboratory studies regarding civil engineering often entail the experimental investigation of materials, structural elements, and scale models to understand and predict their performance under various conditions. For these laboratory studies, precise measurement techniques are imperative, as they provide critical data that can inform the design, construction, and maintenance of infrastructure. Digital image correlation (DIC) is a modern optical method that uses feature tracking and image registration to measure displacement and strain over the whole field of the material or the structural elements. Traditional measurement techniques often involve contact with the specimen and may be intrusive or limited in the measurement points. In contrast, DIC offers high spatial resolution and the capability to capture full-field data, making it highly advantageous for various applications.

**Figure 1:**
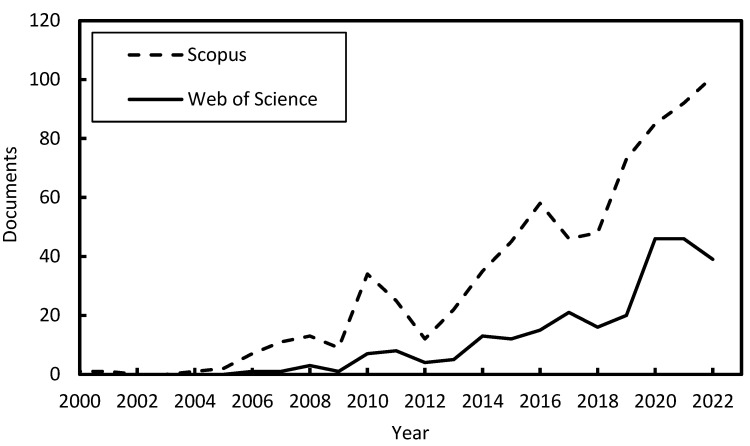
Number of published research articles indexed in Web of Science or Scopus databases using the keywords: “Digital Image Correlation” OR “DIC”) AND (“civil” OR “structure” OR “concrete” OR “beam” OR “steel” OR “concrete beam” OR “steel beam” OR “composite structure” OR “structural joint” OR “column” OR “masonry”.

Since its early development in the 1980s, the DIC methods have found various venues of application in many fields, such as solid mechanics, materials, structures, biology, and others [1,2]. In civil engineering tests, DIC is instrumental in analyzing complex phenomena such as crack propagation, deformation under loading, and strain distribution in a non-destructive manner. The DIC’s full-field and non-contact characteristics encouraged its use in civil engineering tests and applications as a deformation measuring tool. Although DIC has been employed in a wide range of studies, there is a vast and growing body of related literature, making it difficult for practitioners to stay abreast of all developments and applications. Evidence of this spread is expressed in the growing number of scholarly articles exploring the potential of the DIC method in structural and civil engineering. Figure 1 presents the number of documents published in the years between 2000 and 2022 when searching the keywords <DIC> “AND” <Civil> related words retrieved from the Web of Science and Scopus databases. Figure 1 shows the increasing trend in published research over the past 15 years, during which the number of research papers published between 2010 and 2020 more than tripled.

**Figure 2:**
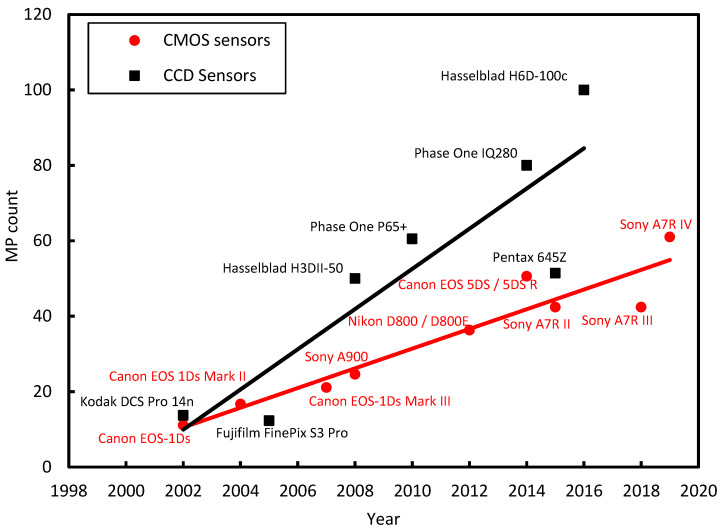
Highest resolution CCD and CMOS cameras over the last 20 years, with the sensor count provided in megapixels (MP).

The acceptance and adoption of the DIC technique are credited to two developments: (1) the advancement of optical instruments, such as digital cameras and scanners, and (2) the rise in the computational capabilities of computers. The advancements in digital cameras, such as resolution enhancements, have facilitated the use of the DIC technique for a broader range of applications. For example, the testing of masonry walls and infills involves coverage of a larger area, which requires a high-resolution camera to provide sufficient spatial resolution. Figure 2 illustrates the progress in regard to the resolution improvement of digital cameras and their sensors over the past two decades. The figure showcases the top-performing sensors, whether CCD or CMOS, for each year within this period.

**Figure 3:**
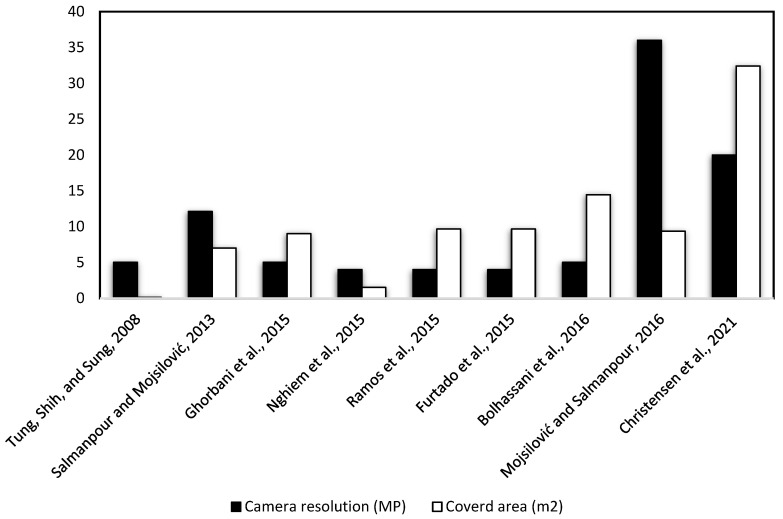
DIC reported areas and camera resolution of masonry and slab tests [4,6,8,9,11,12,13,15,47].

Moreover, Figure 3 demonstrates the correlation between camera resolution and the size of the samples examined. It is evident from Figure 3 that as camera resolution improves, there is an observable increase in the size of the areas investigated during the DIC structural testing of masonry walls and slabs. It is worth noting that these tests utilizing DIC were not feasible until recently [3,4,5,6,7,8,9,10,11,12,13,14,15]. The enhancement of camera resolution has significantly broadened the applicability of digital image correlation (DIC), not only facilitating its use for larger specimens but also extending its utility regarding small and micro-scale specimens. This improvement is attributed to the increase in pixel density, enabling a higher resolution within the same observational area. Consequently, this advancement has made micro-speckling techniques feasible for these specimens, also enabling their ease of application.

The camera hardware boosting not only helped to utilize the DIC testing of large specimens but also assisted in expanding the usage of the technique for various static and dynamic tests. For example, the progressively increasing camera frame rates enabled the use of the DIC for dynamic applications, such as the determination of the structure’s natural frequency, seismic response, vibration measurements, and impact tests [16,17,18,19,20,21,22,23,24,25]. In addition, camera portability, achieved through the use of devices such as smartphone cameras, has opened new venues for DIC applications in civil engineering and other fields [26,27,28,29,30,31,32]. Other factors such as sensitivity improvement, noise reduction, automation, and software integration with techniques such as acoustic emissions (AE) [33,34,35,36,37,38,39], fiber optics [40,41,42], and the recently developed combination of artificial intelligence (AI-DIC) and machine learning (ML-DIC) [43,44,45,46], have all helped to expand the use of DIC in civil engineering lab applications. Thus, the DIC applications in the civil engineering field offer great potential due to their non-contact, full-field, highly accurate, versatile, and real-time monitoring. 

The purpose of this paper is to present a comprehensive review of the uses of digital image correlation (DIC) in civil engineering laboratory experiments. The paper aims to critically analyze the advantages and limitations of DIC and explore potential future directions. It begins with an introductory overview of DIC, followed by a detailed discussion of its applications in various laboratory civil engineering experiments. These experiments include testing concrete beams, columns, pillars, masonry walls, infills, composite materials, structural joints, steel beams, slabs, and other structural members. Additionally, the paper highlights the limitations of DIC in tests involving large or complex setups. A summary of the reviewed works is presented in table format, and the paper concludes with an insightful synthesis of the potential of DIC as a metrological tool for measuring surface deformation and strain of structural members.

## 2. Laboratory Structural Tests and Applications Using DIC

The DIC is applied by researchers in the laboratory to measure the surface deformation and strain of various structural components, as shown in Table 1.

### 2.1. Concrete Beams

Concrete beams come in various sizes and configurations and are one of the most lab-tested components using the DIC technique. Gencturk et al. (2014) reported the DIC’s advantages and limitations after testing a full-scale prestressed concrete structure [53]. The authors used a stereo 3D-DIC for full-field displacement and strain measurements, in conjunction with the conventional displacement transducers. The prestressed I-shaped beam was tested under an ultimate load until failure. It is shown that DIC provided very precise and detailed in-, out-of-displacement, and strains that were impossible to obtain with the conventional measuring methods. However, light sensitivity, measuring surface preparations, data loss due to speckle damage following concrete spalling, and the inability of the DIC algorithm to detect and measure crack width were all listed as limitations when utilizing the DIC to test the concrete beams [53]. 

Alam et al. (2014) applied acoustic emission (AE), in addition to the DIC, to identify the parameters of the fractures, such as fracture zone size, crack openings, and the crack tip [33,39]. The author analyzed geometrically scaled concrete beams to identify and visualize how cracks initiate and form in concrete. The study concluded that combining both techniques provided an efficient and effective tool for measuring fracture and cracking in concrete [33,39]. Similarly, the AE was also used with DIC to investigate the early damage in old-aged RC beams [38]. The investigation proposed combining AE and DIC to verify the damage, where AE delivers an accurate indication of the crack opening, and DIC provides the strain, crack pattern distribution, and local and global deformation of the beam. It highlighted that DIC and AE are suitable for damage detection in existing concrete structures [38]. Similar investigations combined the DIC and AE to identify damage and cracking behaviors in prestressed concrete sleepers of railway structures [34].

DIC was also implemented for crack assessment in large reinforced concrete beams and walls [103]. The author developed a post-processing approach to cope with full-field DIC measurement noise. The suggested approach is validated by testing full-scale and 1/3-scale reinforced concrete beams under four-point bending. The noise filtering strategy was proven effective and provided accurate estimations, including in-plane displacement and strain fields when post-processing the DIC data. The crack widths and patterns were also identified and precisely measured using the suggested approach [103]. 

For the characterization of the shear behavior of fiber-reinforced concrete beams, Gali and Subramaniam (2017) utilized the DIC to monitor full-field displacement and shear cracks [57]. The tested beams measured 125 mm × 250 mm × 1500 mm-(W × D × L), respectively, with 0.5% in volume of 60 mm × 0.75 mm hooked end steel fibers. The DIC test images were captured with a 5 MP resolution camera sensor placed 1 m from the specimens to provide 7–10 pixels per mm. The DIC random error was low after calibration in the range of 0.02 pixels (~0.002 mm). The DIC process was analyzed using VIC-2D software to identify and understand the horizontal displacement and shear crack openings using 80 × 80 pixels subsets [104]. In addition, the experimental results from the DIC were compared with the numerical calculations using ABAQUS software through a user-defined element for the cohesive zone. It was concluded that using the DIC enabled the tracking of the changes in material behavior across the crack after the formation of the shear cracks [57]. 

Suryanto et al. (2017) investigated the shear behavior by employing the DIC to map the cracks on shear-critical RC beams [58]. Three RC beams were tested, one control and two others with stirrup shear reinforcements of 0.4 and 1.1%, respectively. The study investigated the spacing influence on the failure mode, ductility, and overall strength. Open-source DIC software is used to analyze images captured during the four-point bending load [105]. The study offered clearer insight into the longitudinal strain fields and post-cracking response to track crack development using the DIC full-field deformation maps [58]. 

Daghash and Ozbulut (2017) investigated the flexural response of RC beams reinforced with near-surface-mounted NSM fiber-reinforced polymers (FRP) [56]. Five 2.1 m RC beam specimens were prepared and tested under four-point bending monotonic loading. The tests provided full-field displacement, strain maps, and deflection using the DIC, while the FRP strain response was captured using strain gauges. The DIC determined the constant moment zone of each beam to calculate the mid-span deflection, which enabled the plotting of the beam’s principal strain and crack distribution [56]. 

Ghahremannejad et al. (2018) investigated the cracking behavior in concrete beams reinforced with 0.5% and 1% by-volume synthetic fibers [106]. The DIC was used to record the crack widths, locations, and spacing for all specimens tested under four-point bending loading. The results of crack parameters from the DIC were validated while testing a notched (25 mm deep, 5 mm width) 150 × 150 × 500 mm beam tested under three point-bending. The notch crack opening displacement of the beam was measured using a clip gauge transducer. The gauge and the DIC results were in agreement (up to a maximum of 10% difference) [106]. 

The continuous development of the DIC technique led some researchers to try easy methods for surface speckling, such as utilizing the quick response (QR) technology [64]. The study used the QR technology as a random speckle pattern to test an 1800 × 150 × 200 mm RC beam under four-point bending loading [64]. The QR speckle was used in addition to the traditional random speckle pattern for performance comparison and to serve as a means of embedding data into the structural components. The DIC was processed using the open-source Ncorr software version 1.2.0 operated as code within MATLAB R2015a [105,107]. The images were captured using a 24 MP Nikon D5200 DSLR camera equipped with a complementary metal–oxide–semiconductor (CMOS) sensor. The DIC analysis’s findings were contrasted with those of the traditional dial gauge and curvature meters. The moment-curvature response using the QR-based speckle compares well with the conventional dial gauge and machine crosshead results. Other works used the DIC for further testing and verification of concrete beams, such as for flexural behavior and cracking process investigation of 25 years old concrete beams [60], fracture analysis of glass fiber-reinforced cement (GRC) [108], crack width and slip measurements of an RC beam [64], flexural behavior of steel in a fiber-RC beam [62], debonding in FRP and fiber reinforced cementitious matrix (FRCM) retrofitted RC concrete beams [61], and surface crack evaluation of RC elements [46] and polyolefin fiber-reinforced concrete [63].

Finally, the limitations of the conventional 3D-DIC have been addressed by Ref. [52] by proposing a multi-camera MC-DIC for large coral aggregate concrete beams. The suggested DIC setup offers an overlapping field-of-view from the multiple cameras calibrated and synchronized to provide a continuous MC-DIC view. The resulting deformation field is consistent with the strain gauge. The results demonstrate that continuous-view MC-DIC is a valid 3D full-field measuring technique.

### 2.2. Columns and Pillars

Columns are vital structural elements that transfer the weight of the building from its upper stories to its lower stories through compression. As a result, providing full-field strain and crack monitoring for such elements is crucial, as failure could result in disastrous consequences. The DIC can also be used with other techniques, such as fiber optic sensors, to measure strain behavior and profile in reinforced concrete elements [41], i.e., combined distributed optical fiber sensors (DOFS) and digital image correlation (DIC) to monitor reinforced concrete tensile members reinforced steel rebars. The DIC was utilized to determine the concrete’s external strains, while the DOFs were internally planted on the rebar surface to measure the reinforcement strain. The test configuration captured the images for the DIC using a 24 MP camera, capturing an image every 5 s, and then the images were analyzed with GOM correlate software [109]. The ability and performance of both techniques were demonstrated for the internal and external strain monitoring of the concrete and steel of the RC concrete members. The DIC confirms the strain peak’s locations on the rebars by comparing the results from the DOFs with strain distributions on the surface [41]. 

The application of the DIC was not limited to concrete but was also used for highly brittle materials such as glass columns [68]. The research included the testing of multilayer glass columns under central axial compression using 2D-DIC for compression deformation measurement. Although the convenience of the DIC approach has been shown, its inefficiency as a reference method for investigating glass columns results from photo fixation in one plane. As a result, it was recommended to use the 3D-DIC for column elements [66,68]. 

The ultimate axial and lateral strains of preloaded RC circular columns wrapped with FRP sheets were also investigated and derived based on data provided by the DIC [70]. The DIC setup was employed using a VIC-3D system to measure the curved surface of the columns. Eighteen columns with a dimension of 0.20 m diameter × 0.75 m height were prepared, preloaded through heating and cooling (800–1000 C), and then tested under compression loading. The DIC system characterized the columns and the CFRP sheets for deformation and strain. The study concluded that using the DIC technique reveals varied strain distributions along the column height, particularly in pre-heated and confined columns. The results emphasize the DIC values, showing full-field and not-contact boundary-free methods for strain measurement [70]. 

The multiple-camera MC-DIC work proposed by Dai and Li, 2022 was also implemented on large slenderness ratio (0.10 m diameter × 1.80 m height) circular columns [52]. The MC-DIC approach was used to record the 360° view of circular columns under the axial compression test. In the test, 8 industrial cameras with 5 MP resolution and 25 mm focal length were used to cover the mid-span region of the column, namely, 0.10 m × 0.60 m. The MC-DIC has successfully reconstructed the 3D shape of the columns, and both axial and lateral displacement were measured and compared with conventional strain gauges. Similar to the observations of Al-Kamaki (2021), the strain was not evenly distributed along the column’s horizontal and vertical axes, especially in the case of lateral displacement, where the concave side exhibited a larger compressive displacement than the convex side [70]. The strain measurement shows good consistency with the strain gauge data. As a result, the MC-DIC method proved reliable and capable of realizing continuous full-field measurements of large slenderness ratio structural components. 

### 2.3. Masonry Walls and Infills

Masonry walls are often unreinforced and are vulnerable to out-of-plane loading due to their low inertia in that direction. Observing, monitoring, and detecting strain and cracking in such elements is crucial to prevent tipping, infill separation, corner crushing, diagonal cracking, or collapsing. The DIC was thoroughly employed to investigate various physical components, such as displacement, strain, joint motion, drift, and surface bonding to composite and other components of the masonry walls and infills. For example, the full-field measurements of the deformation and the crack mapping of the confined masonry walls were investigated by Ref. [15] using a 3D-DIC setup in addition to conventional pointwise sensors. The DIC was employed to determine three full-scale walls having dimensions of 2.43 m width × 2.49 m height × 0.20 m thickness, which were constructed from hollow concrete masonry units. The 3D-DIC setup faced several challenges, such as high-contrast speckle pattern design and application for large areas of masonry to ensure optimum spatial and measurement resolution balance. The second challenge was the setup calibration required to determine the optimum field of view without sacrificing the spatial resolution. The calibration was performed by manipulating the camera focal length, stereo angle, camera spacing, and camera distance. The third challenge was the stereovision lens distortion correction, requiring pre-calibrated grid panels with known spacing. As a result, two charged couple device (CCD) 5 MP cameras with a 17.6 mm focal length, placed 1.1 m apart over a rigid bar, were placed at a 6.7 m distance from the wall to capture the full-field 3D deformation components. The DIC results of the displacement and the strain maps were evaluated, and an experimental indication of diagonal strut development was presented for the masonry infills. The crack maps were obtained from the 3D-DIC images based on the principal strain maps. The DIC showed a practical approach to hand-marked crack mapping by showing significant details of both the strains and crack maps [15].

Similar work was performed by Ref. [11], where 3D-DIC was employed for large masonry specimen monitoring. In conjunction with the Moire fringe projection technique (for comparison), the DIC was utilized to reconstruct the masonry wall rigid body motion, displacement, and strain measurements in both the in-plane and out-of-plane directions. The in-plane test using three hydraulic cylinders was performed in a 4.22 m × 2.30 m masonry wall encased within an RC frame. Another out-of-plane test was performed on the masonry wall using the DIC with LVDT. The DIC setup includes using two 4.1 MP CMOS sensor cameras with 16 mmm focal length lenses, while the in-plane test includes using only one camera, 2D-DIC since only in-plane components are involved. The DIC images were analyzed using the VIC-3D™ system provided by Correlated Solutions Co. [110]. The 3D-DIC required calibration to measure the intrinsic and extrinsic parameters of the camera. The displacement and strain field values were considered sound after validation with the Moire fringe technique and pointwise measure LVDT tools. The separation between the masonry and the RC frame and corner crushing was also identified using the DIC. However, the diagonal cracking could not be identified because of the wall-side plastering. It is also noted that the DIC application is advantageous, as it provides a full-field, continuous, and easy measuring technique for motion, displacement, strain, and crack mapping. However, the DIC requires a controlled environment for lighting and vibration prevention, requiring trained personnel for operation [11].

The in-plane shear deformation capacity of unreinforced masonry walls was investigated by Ref. [12] by testing ten full-scale masonry walls exposed to in-plane static-cyclic loading. The deformation was measured using pointwise contact LVDT, potentiometers, and the non-contact DIC technique. The DIC setup involved using two 12 MP (70 mm focal length and 0.68 m spatial resolution) DSLR cameras for the preliminary test and 36 MP (50 mm focal length and 0.58 m spatial resolution) cameras for the primary test. The cameras were placed at 5 m and 6 m for the preliminary and primary tests, respectively. The camera placement at such a considerable distance served two purposes: to provide a large field of view for specimen measurement and to reduce the out-of-plane effect on the in-plane measurement when the camera distance is inversely proportional to the out-of-plane error. The applicability of DIC and issues regarding large deformation measurements were discussed and highlighted. The displacement and strain field of the deformed masonry walls were successfully evaluated and quantified. However, the post-cracking phase was not analyzed due to the speckle pattern damage resulting from the concrete outer shell falling off. It also highlighted that the DIC could not completely replace the conventional measurement system due to its inability to deliver high-frequency and real-time measurements. This issue was successfully treated later when computer computational and graphic capabilities were improved. For example, Refs. [48,111,112,113,114] all successfully applied the DIC for real-time displacement and strain measurement applications. The findings showed that an in-house designed DIC system based on standard DSLR cameras could measure the whole deformation field with high accuracy and spatial resolution, even for large specimens and complex deformation fields.

The infilled masonry frame structures were critically evaluated by Ref. [10] by testing several infilled and partially infilled frame specimens exposed to cyclic in-plane loading. The DIC was applied for the displacement and strain assessment of the structure under such loading conditions. No information was provided about the DIC and camera specifications. According to the full-field deformation DIC data, two distinct deformation phases are seen in the masonry infill, one during the hardening stage and another entirely different deformation during the softening phase. Using the DIC method proves that Polyakov’s approach works, but only during the hardening phase of the loading. During this time, the infill masonry panel can be replaced by compression struts because concentrated strain bands can be seen in the wall, while the rest of the structure is only slightly deformed. The “strut” inclination is a significant source of difference between the DIC and the models provided in the literature. The study suggested developing a new macro-model that more realistically describes the softening stage, based on data provided by the DIC. 

Other DIC applications to masonry include but are not limited to, the uniform and non-uniform lateral loading to scaled masonry panels [3], bonding of FRP-masonry bonding [75], FRCM shear behavior [115], semi-interlocking masonry wall cyclic loading [14], masonry damage assessment [4,6], and the microcrack and strain monitoring of brick walls [5,7,8].

### 2.4. Composites Materials

Due to their mechanical characteristics, i.e., environmental resistance (particularly in corrosive atmospheres), fatigue performance, and high weight-to-resistance ratio, composite materials are a viable substitute for conventional steel-based systems in civil engineering rehabilitation and the reinforcement of structural elements. The characterization of the composite material requires quantifying the deformation of the composite components, namely, the matrix and the reinforcement. As a result, the DIC appears to be a promising technique for composite deformation characterization, as it provides a full-field strain and displacement measurement tool. Moreover, the non-contact feature enables the use of the DIC for hard-to-attach specimens; for example, Ref. [93] used the DIC to characterize six fiber-reinforced polymers (FRP) grids tested with direct tensile loading. The study used open-source DIC Ncorr software for image analysis [105]. The data calculated from the DIC, namely the Young’s modulus, were compared with the conventional strain gauge. The DIC setup used an 18 MP camera CMOS sensor to capture an image every 2 s during the tensile load test. The camera field of view was zoomed in due to the narrow width of the grid to increase the spatial resolution of the image and enhance the strain accuracy. Results from the DIC were consistent with the strain gauge data, with less than a 5% difference for all specimens. The study recommended using only the linear average value of Young’s modulus and did not recommend using the strain map directly from DIC analysis, as it showed too many variations. The instantaneous strain variations could be attributed to several factors, such as light fluctuation, bias error, external vibration, and camera movement. Similar to the study in Ref. [93], Ref. [116] studied the characteristics of geogrids utilized in GRS systems and pavement constructions using the DIC technique. A successful measurement of the deformation of the geosynthetics during a tension test using the wide-width strips method was made possible thanks to the use of the 2D-DIC methodology under lab control conditions. The measurement approach also allows for validating models of geosynthetics created using the finite element method (FEM). In addition, the employed method permits the description of the comprehensive surface characteristics of the geosynthetic tested, allowing for the identification of exhausted areas and those outside the base that can affect the strength and displacements of geosynthetic reinforced structures.

The influence of confinement material on the compressive behavior and strain distribution of FRP-confined concrete specimens was studied by Ref. [71]. The experimental investigation involves testing ten confined and unconfined specimens to measure various types of strains. During axial loading, the development of axial, lateral, and Von Mises strains was monitored and investigated, along with the behavior of post-peak strain softening. The data provided by the DIC were validated with contact measuring equipment, LVDT, and a strain gauge. This study’s DIC data demonstrated that the shear zone expansion for unconfined concrete is more localized than for the FRP-confined sample. It was also shown that DIC, compared to contact techniques, yields a more accurate assessment of the final state, owing to its capability to capture the development of full-field strains.

Other composite testing and characterization in civil engineering using the DIC include but are not limited to, the composite additive manufacturing of large-scale composite structures [89], carbon fiber reinforced polymer CFRP tensile testing [91], CFRP-steel composite members bond-slip characterization [69], bond-slip modeling [76], FRP-masonry bonding investigation [75], and composite sandwich structure dynamic response subject to air-blast loading [90]. 

### 2.5. Structural Joints

Welding and bolts are the most widely used connection tools used to join structural steel members due to their flexibility and rigidity. However, thermal and mechanical stresses may occur in the welded or bolted joints during the welding or bolt fabrication. The different properties of the materials may result in heterogeneous mechanical responses surrounding the joint region. Consequently, by providing a full-field deformation map, the DIC may provide extensive data and insight into the weld– and bolt–steel interface regions. For example, Ref. [81] used the DIC during the tensile testing to investigate the local constitutive characteristics of a welded steel joint. The study selected a Q345 steel plate of 20 mm thickness as a welded joint. The welded steel plates were cut into two tensile specimens, with the welding in the center. The samples possessed a cross-section of 25 mm × 20 mm and 100 mm gauge length. The DIC setup included using two cameras to capture the 3D deformation. The cameras were positioned prior to the samples’ mid-section, and the images were captured at a 2 fps sampling rate. Two displacement and strain gauges were also used to capture the sample’s longitudinal deformation and validate the DIC data. The DIC’s estimates of displacement and strain were in good agreement with those obtained from the displacement gauge and strain gauge.

Recently, new software techniques integrated into the DIC have emerged, thanks to the advancements in the application of computer sciences to machine learning, image processing, and computer vision. For example, the DIC was coupled with the artificial neural network to detect anomalies such as the loosened bolts in the beam-to-column joints of a double-story steel frame [45]. The study used a single high-speed Phantom camera (model v341) to record the frame vibration at a frequency of 106 Hz. The DIC measurements were obtained from eight pre-tensioned bolts that are intentionally loosened to detect the connections’ anomaly. The results based on the displacement data from the DIC increased the efficiency of detecting the changes, especially when the algorithm was combined with an artificial neural network (ANN), providing an error of less than 1% for false alarms. 

Similar work was performed by Ref. [83] to capture the in-plane drift of a five-story steel frame subjected to high-intensity shaking. The high-speed Basler Ace cameras were used to observe the speckle patterns applied at every beam-column joint to capture the global response of the structure. The DIC configuration uses a streaming capture technique, which determines the video capture frequency using the Nyquist factor and peak resonant frequency. The camera setup was a 2.3 MP full high-definition resolution camera with a 16 mm focal length, recording images at a maximum rate of 162 fps. The results provided by the DIC were compared with the responses provided by the wire transducers. The study addresses the limitations of the wired transducers compared to the newly configured streamed DIC. As a result, feedback-based control systems may benefit from combining streaming DIC with the template-matching algorithm, since it proved effective in identifying and monitoring various responses.

Other work concerning a steel beam-to-column connection was also investigated [79]. The DIC was used to observe the smallest strain measurements in the connection under controlled laboratory conditions. The strains were measured using the DIC method, and their accuracy was evaluated by comparing the results to those obtained using traditional strain gauges. Finally, the smallest strains that could be reliably measured using the DIC method were determined. Three steel-plate connection specimens were connected by sandwiching an 850 mm steel cantilever beam prone to a single load at its free end. An array of strain gauges was used in addition to the DIC for comparison. The DIC images were captured using a Nikon D800 camera with a 36 MP resolution at a rate of 0.02 fps (an image captured every 30, 60, and 75 s, with the optimum at 45 s). The camera was placed at a very small distance (7.5 to 20 cm, optimum 10 cm) from the connection surface to capture the smallest strain value possible. The strain results from the DIC were determined, and they can accurately measure compressive strains as low as 0.03%. However, a pronounced difference between the strain values provided by the strain gauges combined with the DIC was noted. These differences were attributed to various factors, such as specimen vibration, camera vibration, and strain gauge calibration errors. The DIC technique for joint observation in the lab testing was investigated and highlighted. However, the study suggested the need for improvement, and more work is required to successfully apply the DIC for field applications.

In addition, steel is not the only material making up the joints. For example, the CFRP laminate hybrid joint was investigated for damage evolution and failure through single-lab direct tensile testing [82]. With the use of the acoustic emission (AE) method for damage evolution capturing, the DIC was used to examine the full-field displacement and strain in simple bonded and bolted joints. Both two- (180 mm focal length) and three- (50 mm focal length) dimensional DIC, using two Grasshopper CCD cameras at a rate of 5 fps, were employed. Numerical modeling was also used to validate the DIC and AE’s experimental data and the data provided by wired strain gauges, which were impeded in the bolts. 

Other joint-DIC applications include but are not limited to, strain distributions in bolted steel elements [86], epoxy adhesive joint fracture behavior characterization [117], hybrid bonded-bolted composite joints [84], and metal laminate joints [85].

### 2.6. Steel Structures 

Steel beams and girders compose essential structural members in bridges and steel structures. As a result, measuring the full-field deformation to understand the structural behaviors, such as strain, deflection, and curvature, is essential. For example, the curvature of the steel beam was investigated by Ref. [50] using the DIC. The level of accuracy of the DIC curvature measurement technique was investigated by testing a hollow structural section (HSS) steel beam under three-point bending in a controlled laboratory setting. Two Canon T2i cameras with 180 mm focal lenses were placed 1.6 m from the beam face to deliver a spatial resolution of 0.036 mm/pixel. Three strain gauges were placed at the beam’s top and bottom face beside the DIC for further strain monitoring and validation using DIC data. The beam curvature from the DIC showed a similar linear trend and slope to those of the strain gauges. However, a consistent discrepancy existed between the measured curvature from the DIC and the foil gauges. An initial out-of-plane beam displacement relative to the camera before the first load might explain the offset. Because of this, a second DIC analysis was performed, this time with the averaged picture from the first load stage serving as the reference image to correct for the out-of-plane effect. The modified strain profiles for the chosen gauge lengths provide a decent fit in the curvature predictions for the DIC virtual and the physical strain gauges.

DIC is used to analyze an adhesively bonded double-cantilever beam and to calculate a cohesive-zone model traction-separation law [80]. DIC is utilized to identify both the cohesive zone and the elastic arm evolution, according to loading and the adhesive. The double cantilever beam was made of 4130 steel sheets having a thickness of 4.8 mm and a length of 152 mm. Two 5 MP cameras were synchronized and utilized to provide images for the DIC VIC-2D software [104]. The results from the DIC were employed for obtaining the traction-separation law through obtaining the loading points rotation and the J-integral. In addition, shear strains and cohesive zone deformation were also explored using the full-field data provided by the DIC. By establishing a correlation between the cohesive-length scale and the growth of the cohesive zone and the root rotation, it was demonstrated that these two features scale in a remarkable way, which is in line with the analytical calculations of an elastic foundation model. In summary, the full-field deformation data offered by the DIC provides a better cohesive zone development model.

In addition, experimental tests have been conducted to assess the applicability of DIC for deflection measurement of large wide-flange steel beams [95]. The tested beam was made of SS400 steel, 200 mm in width, 8 mm in thickness, and 5 m long, tested under a three-point bending load. The images were recorded for DIC analysis using a single DSLR camera with an 8 MP resolution. A new method was developed for DIC data correction based on camera movement correction by capturing undeformed reference surfaces within the camera field. Thus, the camera was moved and positioned in the same place to verify the applicability of the proposed method. Using displacement transducers, the beam deflection was measured at multiple positions, namely, ¼, ½, and ¾ of the beam length. Then, based on DIC data, the deflection was extracted, corrected, and compared with the transducers’ displacement. The deflection curves after DIC data correction are in good agreement with the deflection from the gauges.

Recently, a new method based on off-axis DIC was proposed by Ref. [118] using a video deflectometer beside the DIC camera. The new method calculates all the bridge points of interest, with a scale factor based on the assumption of the spatial straight-line fitting. The proposed full-field bridge deflection measurement method was tested by applying a static load on a thin 950 mm long cantilever beam to verify the method’s accuracy. The distance between the video deflectometer and the steel support beam is one meter. The lens has an 8 mm focal length, and the deflectometer has an 11-degree pitch angle. A set of dial gauges was placed on the new proposed beam at multiple locations for deflection measurement and to verify the new off-axis DIC method. The benefits of the proposed full-field bridge deflection measurement over the traditional single-point measuring method are: that only the distances to the camera are required, and the 2D-DIC analysis is demonstrated to be accurate and efficient. The newly proposed method proved to be a robust optical tool for measuring bridge deflection. Other DIC applications include the mechanical characterization of a three-point bending of a simply supported W-steel beam [96] and the dynamic analysis and damage detection of a multi-story steel frame [16].

### 2.7. Slabs and Other Members 

Advanced monitoring systems are necessary to identify stop criteria during the proof-loading of concrete slab bridges. Recently, Ref. [47] investigated the standard measuring technique of crack identification and assessment using the 2D DIC technique. The DIC capabilities were explored by testing three overturned T-beam RC slabs under controlled laboratory conditions. Two of the concrete slabs were stripped from an in situ full-scale Ot-slab bridge with a 9 m span and one downscaled slab. Two cameras were used for DIC imaging, one with 20 MP placed 3.8 m from the bridge surface. The other camera was placed closer, at 2.6 m, and had an 18.7 MP resolution. The concrete slab test setup included a pin supported from three edges (two short-span at 1.6 m, and one long-span, at 7.5 m) and one long free edge, and the load actuator was placed in the middle of the long span and 0.5 m from the free edge. The test setup consisted of LVDT, distance lasers, potentiometers, strain gauges, and 2D-DIC. The DIC data were analyzed using GOM correlate software [109]. The crack width measurements were successfully acquired using the 2D-DIC images after out-of-plane movement correction. The load-deflection and crack widths from the DIC agree well with the data provided by the pointwise sensors. As a result, the DIC technique seems suitable for identifying the stop criteria in the proof-loading of the in situ bridge section. 

In addition, the DIC was also used for various qualitative and quantitative civil engineering tests and applications. For example, the shear push-off testing of seven double-L shape RC specimens was investigated by Ref. [100] by employing the DIC technique. The crack kinematics were observed using the DIC’s full-field strain and displacement data. This research proposes a cheap and easy method to add quantitative measures to regular visual inspections by processing images captured with a portable digital camera using open-source DIC software. Two separate cameras were implemented during the test, one primary having an 18 MP and the secondary camera having a 24 MP resolution. A fixed rectangular aluminum frame reminiscent of that used by Refs. [111,119,120] was used as a reference for the DIC data to compensate for the movements involved during the test. During the test, four camera movement types were considered: (1) a camera placed parallel to the structure shifting, (2) camera movement toward the structure, (3) vertical camera rotation, and (4) camera titling, with a horizontal rotation. The study concluded that a significant error was observed for the uncorrected DIC measurements attributed to the camera movement. However, the proposed correction enhanced the experimental measurements, where 96 and 99% of the residual errors were within 0.1 mm of the corrected slip and crack width, respectively. 

## 3. Summary of the DIC-Civil Engineering Laboratory Tests and Applications

A summary of the reviewed DIC tests and applications is presented in Table 2. Table 2 shows the author’s name, year of publication, the specimen type, the test method, the measured parameters, and the camera specification, such as sensor resolution (mega pixel—MP) and measurement resolution ($\frac{Pixel}{mm}$ − px/mm), as well as the DIC software used for the analysis. 

**Figure 4:**
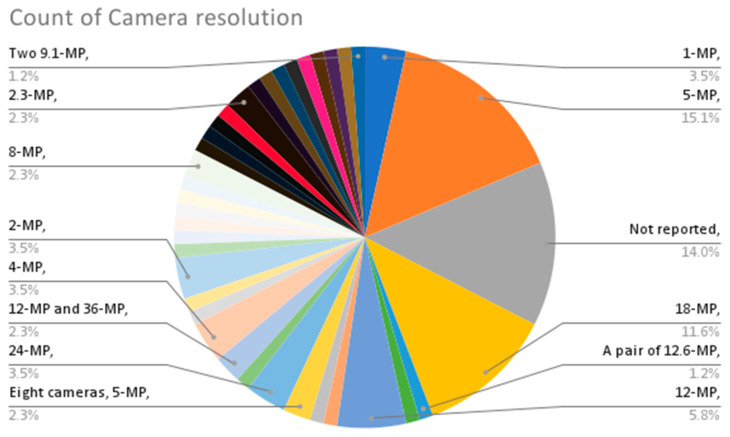
Count of camera resolutions of the reviewed DIC studies.

**Figure 5:**
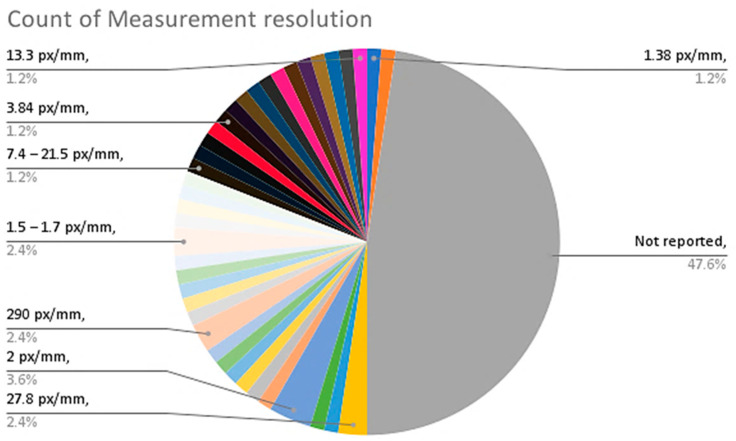
Count of measurement resolutions of the reviewed DIC studies.

**Figure 6:**
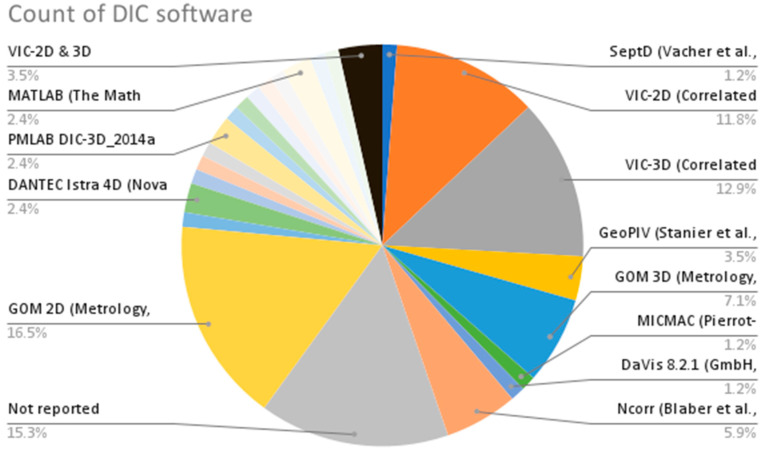
Count of DIC software types used in the reviewed studies.

**Figure 7:**
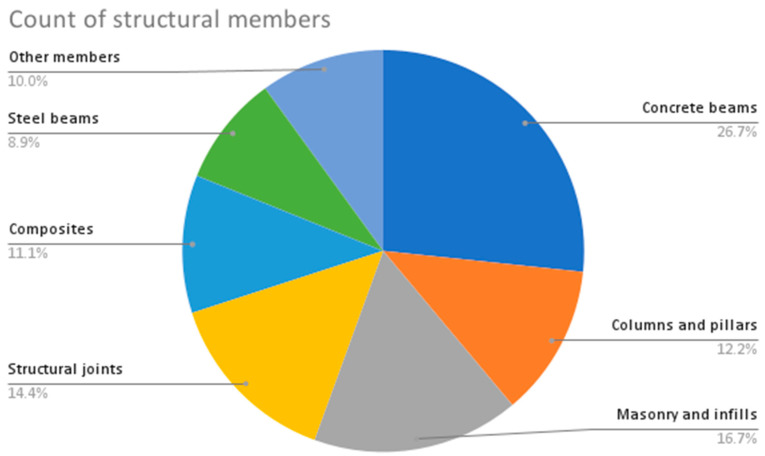
Count of specimen types of the structural members of the reviewed DIC works.

**Figure 8:**
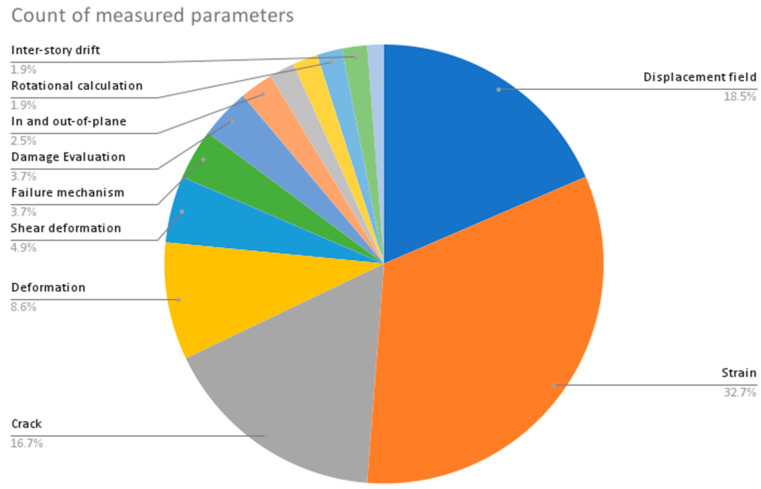
Counts of the measured parameters of the reviewed DIC studies.

**Figure 9:**
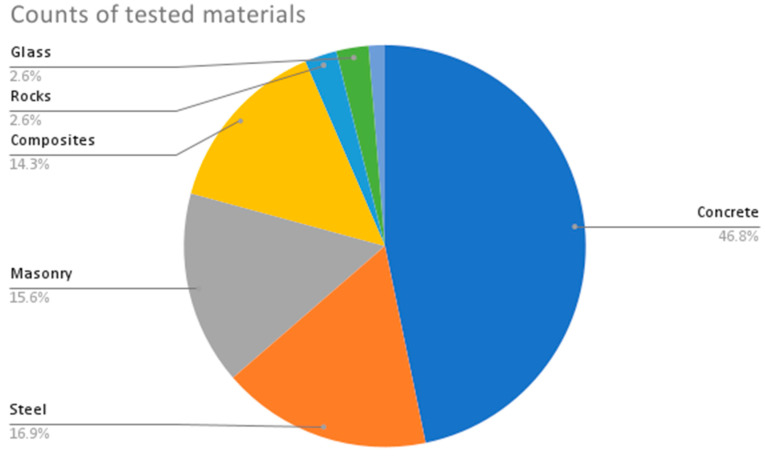
Counts of the tested materials of the reviewed DIC studies.

**Figure 10:**
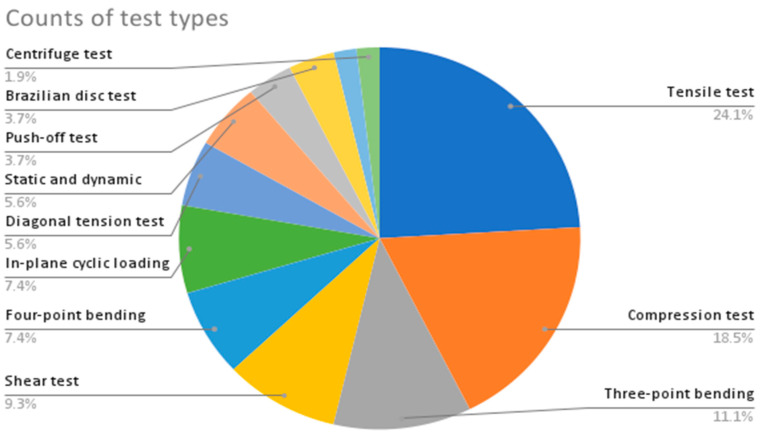
Counts of types of tests used in the reviewed DIC tests.

**Figure 11:**
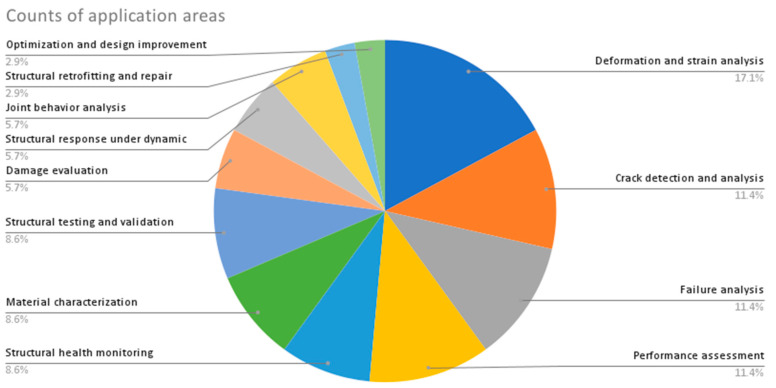
Count of the application areas used in the reviewed DIC studies.

From the provided data in Table 2, the following are some interesting patterns, trends, and statistics that can be observed:

1. Camera Resolution: The camera resolution used in the studies ranges from 0.3 MP to 29 MP, with a majority of studies using cameras with resolutions between 12 MP and 18 MP. Figure 4 shows the camera resolution count of the reviewed studies.
2. Measurement Resolution: The measurement resolution varies widely across the studies, with values ranging from 0.5 px/mm to 290 px/mm. The majority of studies exhibit measurement resolutions between 1 px/mm and 30 px/mm. Figure 5 shows the camera resolution count of the reviewed studies.
3. DIC Software: Several DIC software packages were used, including VIC-2D, VIC-3D, GOM 2D, GOM 3D, GeoPIV, Ncorr, DANTEC Istra 4D, and MATLAB. These software packages provide different capabilities for analysis and measurement. Figure 6 shows the DIC software count of the reviewed studies.
4. Specimen Types: The studies cover a wide range of specimen types, including RC beams, concrete prisms, masonry walls and infills, composite materials, structural joints, steel structures, and slabs. This reflects the diverse applications of DIC in structural testing. Figure 7 shows the count of the specimen types of the structural members of the reviewed studies.
5. Measured Parameters: The studies focus on measuring various parameters such as displacement fields, strain distribution, crack width, deformation, fracture parameters, and failure mechanisms. This demonstrates the versatility of DIC in capturing different aspects of structural behavior. Figure 8 shows the counts of the measured parameters of the reviewed studies.
6. Material Types: Different materials were tested, including concrete, steel, masonry, composites (such as FRP and GFRP), and geosynthetics. This highlights the applicability of DIC across a range of materials used in structural engineering. Figure 9 shows the counts of the tested materials of the reviewed studies.
7. Test Methods: Various test methods were employed, such as three-point bending, four-point bending, compression tests, tensile tests, shear tests, uniaxial loading tests, and cyclic loading tests. This indicates the flexibility of DIC in accommodating different testing scenarios. Figure 10 shows the count of the test types used in the reviewed studies.
8. Application Areas: The studies cover a wide range of application areas, including the monitoring of structural health, the evaluation of material properties, the detection of damage and cracks, the assessment of performance under different loads, and the analysis of failure mechanisms. Figure 11 shows the counts and percentages of the application areas.

These patterns, trends, and statistics provide insights into the diverse use cases and capabilities of DIC in structural testing and analysis.

## 4. Conclusions

The use of digital image correlation (DIC) in structural testing and analysis has showcased its versatility and capabilities. The studies employed cameras with resolutions ranging from 0.3 MP to 29 MP, with a majority falling between 12 MP and 18 MP. The measurement resolutions varied widely, from 0.5 px/mm to 290 px/mm, with the majority falling between 1 px/mm and 30 px/mm. Different DIC software packages, such as VIC-2D & 3D, GOM 2D and 3D, etc., were utilized for analysis and measurement. The studies employed a diverse range of specimen types, including RC beams, concrete prisms, masonry walls and infills, composite materials, structural joints, steel structures, and slabs. The measured parameters encompass displacement fields, strain distribution, crack width, deformation, fracture parameters, and failure mechanisms. The following conclusions, based on each type of structural member, can be drawn:

1. Concrete beams: The DIC is proven to be suitable for damage detection in existing concrete beams, monitoring shear behavior, measuring crack development, and assessing flexural response. The development of new speckling methods, such as Quick Response (QR) technology, improves DIC’s surface preparation. Additionally, multi-camera MC-DIC provides an innovative solution for members with a high slenderness ratio. The MC-DIC provided a continuous and valid 3D full-field measuring technique for large concrete beams.
2. Columns and pillars: The DIC has been successfully applied for monitoring strain and crack behavior in columns. Also, the DIC can be combined with other methods, such as the used of fiber optic sensors. It has been successfully applied to monitor compression, tensile members, and glass columns, providing valuable information regarding strain distribution. The use of DIC in preloaded columns wrapped with FRP sheets revealed varied strain distributions and highlighted the advantages of full-field and non-contact boundary-free strain measurement tools like the DIC. The implementation of multi-camera MC-DIC demonstrated its effectiveness in reconstructing the 3D shape of circular columns and measuring axial and lateral displacement, showing good consistency with the results of strain gauge data.
3. Masonry walls and infills: The DIC has been successfully applied to study a wide range of masonry walls and infills under various loading conditions, including in-plane and out-of-plane loading, cyclic loading, and damage assessment. The main advantages of DIC are its ability to provide full-field measurements, its non-contact nature, and its high spatial resolution. However, DIC also has some limitations, such as its requirement for a controlled environment, especially for large structures, for which special care is required for the test setup during the DIC application.
4. Composite materials: The DIC emerges as a promising technique for characterizing composite deformation, providing full-field strain and displacement measurements. The studies in this field highlight successful applications of DIC in characterizing various composite materials, such as fiber-reinforced polymer (FRP) grids and geosynthetics, showcasing its accuracy in comparison to conventional methods like the use of strain gauges. Additionally, the review emphasizes the importance of using DIC for assessing the tensile behavior and strain distribution in FRP-confined concrete specimens, noting its superiority over contact techniques for capturing full-field strains.
5. Structural joints: DIC proves effective in providing full-field deformation maps and has been successfully employed in various studies, including investigating the local constitutive characteristics of welded steel joints and detecting anomalies like loosened bolts in steel frame connections. The integration of DIC with advanced software techniques, such as artificial neural networks and template-matching algorithms, enhances its capabilities for detecting changes and monitoring structural responses. However, challenges like vibration, calibration errors, and the need for improvement in field applications are acknowledged. Additionally, the application of DIC extends beyond the evaluations of steel joints, as seen in the examination of CFRP laminate joints, revealing its versatility for analyzing different materials and joint configurations.
6. Steel beams: DIC has been employed to measure full-field deformation, including strain, deflection, and curvature, providing valuable insights into structural behavior. Researchers have investigated the accuracy of DIC curvature measurement techniques and have achieved results consistent with those obtained from strain gauges after correcting for initial out-of-plane displacement. Additionally, DIC has been utilized to analyze adhesively bonded double-cantilever beams and obtain the cohesive-zone model’s traction-separation law, contributing to the development of cohesive zone models. Experimental tests have shown the applicability of DIC for the deflection measurement of large wide-flange steel beams, with good agreement between the DIC data and transducer displacement after correction.
7. Slabs and other members: Recent research investigated the standard crack identification in slabs using 2D DIC, showing successful acquisition of crack width measurements and agreement with pointwise sensor data. Additionally, DIC has been utilized in various civil engineering tests and applications, such as shear push-off testing of double-L shaped RC specimens, allowing for the observation of crack kinematics, such as crack mouth opening displacement (CMOD), through full-field strain and displacement data. This study proposed a method to add quantitative measures to visual inspections using open-source DIC software and emphasized the importance of correcting for camera movement to enhance experiment measurements, ultimately enhancing the accuracy of the results.

The DIC proved applicable to various materials used in structural engineering, including concrete, steel, masonry, composites (such as FRP and GFRP), glass, and geosynthetics. Different test methods, such as three-point bending, four-point bending, compression tests, tensile tests, shear tests, uniaxial loading tests, and cyclic loading tests, were employed. DIC reveals applications in structural health monitoring, evaluating material properties, detecting damage and cracks, assessing performance under different loads, and analyzing failure mechanisms. These findings highlight the broad range of use cases and capabilities of DIC for use in structural testing and analysis.

## Figures and Tables

**Table 1:** A summary of recent laboratory digital image correlation (DIC) research studies conducted in the field of civil engineering testing.

No.	Structural Element	Authors
1	Concrete beams	[38,39,40,48,49,50,51,52,53,54,55,56,57,58,59,60,61,62,63,64,65]
2	Columns and pillars	[16,17,52,66,67,68,69,70,71,72,73]
3	Masonry and infills	[3,4,5,6,7,8,9,10,11,12,13,14,15,74,75]
4	Structural joints	[17,45,76,77,78,79,80,81,82,83,84,85,86]
5	Composites	[69,71,75,87,88,89,90,91,92,93]
6	Steel beams	[16,18,50,80,94,95,96]
7	Other members	[18,44,97,98,99,100,101,102]

**Table 2:** Summary of the recent lab DIC applications for civil engineering tests.

Author	Specimen Type and Test Method	Measured Parameters	DIC Specifications (Camera Resolution, Measurement Resolution, DIC Software)
1. Concrete Beams			
[60]	Full-scale RC beam after 25 years of service in an industrial environment, four-point bending.	Displacement fields; cracking process.	1-MP, 1.38 px/mm, SeptD [121]
[37]	Plain concrete prisms 840 mm long, 100 mm wide, and 100 mm high; a three-point bending and a crack mouth opening displacement test.	Visualization of the crack opening and strain monitoring.	5-MP, 20 px/mm, VIC-2D [104]
[36]	RC beams with a length of 2.5 m, a distance between the supports of 2.3 m, and a height and width of 0.3 m and 0.2 m, respectively; four-point bending test.	Measuring the displacement and deformation fields at the side and the bottom surface of the beams.	Not reported, Not reported, VIC-3D [110]
[50]	Two different sections and materials, i.e., a steel HSS and a series of RC beams; three-point bending test.	Longitudinal strains with the height at a section (curvature).	18-MP, 27.8 px/mm, GeoPIV [122,123,124]
[53]	Full-scale deep I-beam prestressed concrete beam; shear capacity test.	In-plane and out-of-plane strains; tensile and compressive strain locations.	A pair of 12.6-MP, Not reported, GOM 3D [109]
[33,39]	Geometrically scaled concrete beams under a bending test.	Fracture parameters, such as crack openings and fracture zone size	1.4-MP, 5.5–28.5 px/mm, VIC-2D [104]
[34]	Prestressed concrete sleepers; three-point bending test.	Critical damage area evolution as a function of loading periods	5-MP, 7.1 px/mm, VIC-3D [110]
[103]	RC beams: 1. full-scale rectangular beams; 2. one-third scaled mock-ups; four-point bending.	Evolution of the crack pattern; crack width, deformation, and strain monitoring.	12-MP, 2 px/mm, MICMAC [125]
[65]	RC beams with both small and large crack slip; three- and four-point bending tests.	Crack width and crack slip measurement.	18-MP, 7.5–21 px/mm, GeoPIV [122,123,124]
[62]	Normal strength, high strength concrete, and high strength fiber concrete, with nine RC beams tested under four-point bending.	Strain, crack detection, development, and width measurements.	Not reported, Not reported, GOM 3D [109]
[61]	Composite-retrofitted RC beams; four-point bending.	Strain and displacement monitoring, cracking, and debonding.	5-MP, 4.6 px/mm, DaVis 8.2.1 [126]
[57]	RC beams reinforced with steel fibers; beam dimensions: 1500 mm length, 125 mm width, and 250 mm depth; four-point test; beams with a shear s/d ratio = 1.8.	Cracks and crack pattern identification;tracking the response of the concrete across a crack; horizontal and vertical displacements.	5-MP, 7–10 px/mm, VIC-2D [104]
[58]	Three RC beams, two with shear and one without shear reinforcement, with dimensions: 100 × 150 × 2000 mm^3^; four-point loading test.	Crack mapping; longitudinal strain fields.	18.4-MP, 4.4 px/mm, Ncorr [105]
[56]	Five RC beams, strengthened with NSM-BFRP, with a length of 2.1 m; four-point bending test.	Full-field strain and displacement contours; mid-span deflection; measurement of crack width.	5-MP, Not reported, Not reported
[106]	Fiber-reinforced concrete beams, six 1143 × 229 × 152 mm with and six 507 × 152 × 152 without, longitudinal reinforcement; four-point test.	Crack kinematics (location, number, spacing, and width).	Not reported, Not reported, GOM 2D [109]
[64]	A total of 24 (RCC) beams of 1800 mm × 150 mm × 200 mm in size.	Obtaining the moment (M)—curvature (κ) relationships.	24.1-MP, Not reported, Ncorr [105]
[46]	Post-tensioned, precast crane runway I-section beams, with dimensions of 6 m in length and 0.8 m in height, after more than 50 years of use; three-point bending test.	Full-field strains;detecting and locating the cracks on the surface; combining DIC with R-CNN.	Not reported, Not reported, μDIC [127]
[128]	RC beam with dimensions of 4 × 0.3 × 0.15 m; four-point bending test.	Strain field.	Not reported, Not reported, Ncorr [105]
[38]	Full-scale RC beam used for 60 years in an industrial environment; four-point bending test.	Strain, crack pattern and distribution, global and local deformation.	5-MP, 7.7 px/mm, DANTEC Istra 4D [129]
[63]	Polyolefin fiber RC beam; three-point bending fracture test results.	Crack width; crack opening displacement plane.	5-MP, 200 px/mm, Not reported
[52]	Coral aggregate concrete beam with dimensions of 1350 mm × 200 mm× 120 mm; four-point bending.	A continuous-view multi-camera DIC to continuously measure the full-surface deformation.	Eight cameras, 5-MP, 290 px/mm, Not reported
[130]	Four concrete beams (150 × 150 × 600 mm) tested under four-point bending load.	Displacement fields;crack propagation.	18-MP, Not reported, Py2DIC [131]
[132]	A 1.4 m long inverted prestressed T-beam was tested under four-point bending.	Longitudinal compressive strain profile.	12-MP, 10 px/mm, GOM 3D [109]
2. Columns and Pillars			
[66]	Multilayer glass and polymeric film sheet columns with 1000 mm height, 5 × 10 mm sheet thickness, and 70 mm width; compression test.	Determination of the relative deformation and the strain of the glass.	18-MP, Not reported, GOM 2D [109]
[41]	Three types of RC tie members tested under uniaxial tensile loading with the samples clamped to the testing machine using the reinforcing bars.	Determination of the surface displacements and strains of the columns.	24-MP, 7.5–10 px/mm, GOM 2D [109]
[68]	Two series of glass columns with 70 × 100 and 35 × 100 sections, both 1 m in height; all samples were tested under compression loading.	The use of 2D-DIC for glass columns under compression is not possible because the columns are deformed in the direction of the three axes in space. For this study, it is necessary to use 3D-DIC.	18-MP, Not reported, GOM 2D [109]
[70]	Pre-loaded RC columns loaded under heating and cooling, then repaired with CFRP sheets; compressions test.	Determination of both lateral and axial surface strains of the undamaged and post-heated CFRP-confined columns.	5-MP, 1.1 px/mm, VIC-3D [110]
[52]	Circular timber slender columns, 100 mm in diameter and 1800 mm in height were tested under compression loading.	Full reconstruction of the 3D shape of the circular column.	Eight cameras, 5-MP, 290 px/mm, Not reported
[73]	A precast concrete column reinforced with steel-FRP composite bars; six columns (300 × 360 × 1400 mm) were tested under low reversed cyclic loading.	Strain distribution; moment-curvature curves; plastic hinge region; rotation calculation.	Not reported, Not reported, Not reported
[133]	Concrete column under controlled load with a vertical stress of 2.4 MPa under compression.	Displacement and strain field.	12-MP, 22 px/mm, Global DIC and 7D [121]
3. Masonry Walls and Infills			
[8]	A 30 × 40 cm 45° brick wall and a 150 × 120 cm steel-framed brick wall, both tested under compression load.	Displacement and strain measurement;crack observation	6.3-MP, 5 px/mm, Not reported
[3]	Several 1/6th scale masonry wall panels, using a centrifuge to correctly model the self-weight, with uniform lateral loading using airbag or non-uniform hydraulic loading.	Deflection of the panels; identification of cracks in the wall panel.	Not reported, Not reported, Not reported
[13]	Eleven full-scale unreinforced masonry walls, ranging from 1.5 × 1.6 m to 3.6 × 2.6 m, were subjected to in-plane cyclic loading.	Deformation, strain, and crack distribution.	12-MP and 36-MP, 1.5–1.7 px/mm, VIC-2D [104]
[10]	One single-bay infill, one single-bay partially infilled frame, a two-bay partially infilled frame, and a squat infilled frame; all were subjected to cyclic lateral loading.	Strain fields; strut inclination.	Not reported, Not reported, CORRELI-Q4 [134]
[15]	Three full-scale masonry walls with dimensions of 2.4 × 2.4 m, were confined in an RC frame; all specimens were subjected to lateral in-plane cyclic loading.	drift and diagonal deformation fields;slip at the interface between the masonry and the concrete tie column.	5-MP, 0.72 px/mm, VIC-3D [110]
[6]	A large-scale physical model reproducing both the soil-structure interaction and the masonry structure. The loading is applied by means of ground surface displacement.	Proposing a method of quantification of DIC measurement errors; reconstruction of motion displacement fields; damage evaluation.	4-MP, 7 px/mm, VIC-3D [110]
[9]	A full-scale 4.2 × 2.3 m masonry wall confined with an RC frame. Two tests were applied: one in-plane shear test and a quasi-static out-of-plane cyclic test.	Displacement and strain fields; principle strains; out-of-plane displacement.	4-MP, 2 px/mm, VIC-3D [110]
[11]	A full-scale 4.2 × 2.3 m infilled RC frame subjected to in-plane testing, with one double leaf-panel.	Diagonal cracking; corner crushing; shear-friction failure.	4-MP, 2 px/mm, VIC-3D [110]
[4]	A full-scale 3.8 × 3.8 m partially grouted masonry shear wall tested under constant vertical compression loading and horizontal lateral loading using a quasi-static displacement procedure.	Crack patterns; diagonal tension and compression strains; bed joint shear; base strain.	5-MP, 0.5 px/mm, GOM 3D [109]
[12]	Ten full-scale unreinforced masonry walls, ranging from 1.5 × 1.6 m to 3.6 × 2.6 m, were subjected to in-plane cyclic loading.	Deformation, strain, and crack distribution.	12-MP and 36-MP, 1.5–1.7 px/mm, VIC-2D [104]
[5]	Brick masonry prisms with dimensions of 520 × 220 × 100 mm were tested under uniaxial compression loading.	In-plane strains and displacement.	12-MP, Not reported, Ncorr [105]
[7]	Six 710 × 710 mm brick masonry specimens were tested using diagonal tension tests to evaluate the shear behavior.	Identification of the strain distribution at the moment of failure; deformation measurement; shear modulus.	24-MP, 100 px/mm GOM 2D [109]
[14]	Different types of engineered masonry panels (2 × 2 m), made of semi-interlocking masonry (SIM) units, were subjected to in-plane cyclic loading.	von Mises strain fields; joint opening and propagation; horizontal and vertical displacement.	8-MP and 36-MP, 7.4–21.5 px/mm, VIC-2D [104]
[135]	Four full-scale masonry infill models (3.1 × 3.0 m), surrounded by an RC frame, were tested against out-of-plane loading.	Full-field out-of-plane deformation and strain concentration analysis.	12-MP, Not reported, GOM 3D [109]
4. Composite Materials			
[90]	GFRP sandwich structure, with a speckled target of 1600 × 1300 mm, subjected to a blast load.	Deformation monitoring; failure mechanism.	Two high-speed cameras of 1000FPS, 1-MP, 0.787 px/mm, GOM 3D [109]
[75]	FRP-masonry composite is used to characterize the bonding interface through tensile and shear tests.	Longitudinal strain distribution in tensile testing; strain distribution along the bonded length in shear testing.	2-MP, 27 px/mm, GOM 2D [109]
[69]	CFRP-steel composite structure with dimensions of 150 × 150 × 500 mm.; shear test to monitor the bond-slip, and compression test to monitor the buckling location.	Derivation of the bond–slip relationship between the CFRP-steel interface. The precise location of the buckling and delamination of a CFRP-steel composite.	A multi-camera system, 4-MP and 5-MP, Not reported, PMLAB DIC-3D_2014a [136].
[115]	Bond and tensile tests were conducted on composite reinforcements comprising different textiles and matrices.	Damage pattern (crack location and width); load transfer mechanism between composite-to-substrate.	24-MP, 9 px/mm, Two types of software: CivEng Vision [137] and Ncorr [105]
[91]	Several DIC (2D and 3D) methods were employed using tensile tests to characterize 25 CFRP composite specimens.	The Young’s modulus; the Poisson ratio; displacement and strain.	Two systems: 2D: 18-MP, 3D: two 18-MP, Not reported, 2D: Ncorr [105], 3D: MultiDIC [138]
[116]	Twenty specimens of 20 × 30 cm of geosynthetics geogrid composites were tested under uniaxial tensile loading.	Displacement and strain distribution; maximum principal strain	Not reported, Not reported, Not reported,
[71]	Ten FRP-confined concrete composite specimens were tested under uniaxial compression load.	Axial, lateral, and von Mises strains.	2.8-MP, Not reported, VIC-3D [110]
[92]	Review of static and dynamic tests of a large-scale composite structure under various loadings, such as buckling, crash, rotating, impact, and fatigue loads.	Full-field deformations; displacement field; dynamic measurements.	3.2-MP, 4-MP, 5-MP, 12-MP, 3.84 px/mm, GOM 2D & 3D [109]
[89]	A Big Area Additive Manufacturing(BAAM) system using a printedfull-size wall with a building envelope of 6.1 × 2.4 × 1.8 (length × width × height).	Thermal residual stresses monitoring.	12.2-MP, 13.6–32 px/mm, VIC-2D [104]
[93]	Six FRP grid specimens consisting of BFRP and CFRP composites, 300 mm in length, were tested under uniaxial tensile loading.	Young’s Modulus; vertical strain.	18-MP, Not reported, Ncorr [105]
[139]	A 500 mm long, 90 mm wide, and 7.5 mm thickness steel plate with an edge crack, repaired with a CFRP sheet, is subjected to uniaxial tensile loading.	Displacement field; crack growth propagation trajectories.	8-MP, Not reported, VIC-2D [104]
5. Structural Joints			
[86]	Bolted steel connection specimens tested under uniaxial tensile loading.	In-plane displacement distributions.	0.78-MP, Not reported, MATLAB [107]
[79]	A steel beam-to-column connection specimen acting as a cantilever loaded at its free end.	Minimum measurable strains at six locations at the joint.	36.3-MP, Not reported, GOM 2D [109]
[81]	Two 25 × 20 sections of welded steel joints, 20 mm thick, were tested under uniaxial tensile loading.	Strain distribution.	Not reported, Not reported, PMLAB DIC-3D_2014a [136].
[76]	Interfacial behavior between a reinforcement material and a substrate was evaluated through a series of single-lap shear tests.	Slip distribution.	18-MP, Not reported, GOM 2D [109], Ncorr [105]
[117]	Adhesively bonded single lap joints tested under tensile and shear loads.	Shear strain distributions; strain distributions in the adhesive layer.	Not reported, Not reported, GOM 2D [109]
[45]	Pretensioned bolted beam-to-column connection anomalies were detected during the frame’s vibrations caused by harmonic excitation.	Damage and anomaly detection in the connections.	1-MP, 15 px/mm, DANTEC Istra 4D [129]
[17]	A downscaled model of RC structure subjected to seismic vibrations.	Biaxial deformation at the beam-column region; displacement and angle of inter-story drift.	0.3-MP, Not reported, MATLAB [107]
[78]	A double cantilever beam (DCB) with aluminium alloy substrate was tested with a screw-driven tensile testing machine.	Crack length measurement; crack tip separation; beam rotation; energy release rate.	18-MP, 28.5 px/mm, GOM 2D [109]
[84]	A single-lap hybrid bonded-bolted (HBB) joint with a bolted hole in the center; the samples were tested under uniaxial loading.	The shear strain around the bolt; surface failure location identification.	1-MP, 30.7–31.9 px/mm, MatchID [140]
[83]	A five-story steel-frame braced structure with a 6 m height and a 2 × 2 m plan area, subjected to bi-directional seismic excitations.	Displacement, acceleration, and velocity time history of the beam-column joint; inter-story drift; strain in joints and floor.	8 high-speed cameras, 2.3-MP, 2.2 px/mm, VIC-2D & 3D [104,110]
[85]	Bolted fiber metal laminate joints consisting of three layers of 0.4 mm thickness T3 aluminium and four layers of GFRP. The samples were tested under uniaxial loading.	Displacement distribution; compression and tension damage dominant regions.	2.3-MP, Not reported, VIC-3D [110]
[82]	Three test coupons for each of the bonded, bolted, and hybrid joints were tested under uniaxial tensile loading.	Strain field of the joint; strain field over the adhesive layer along the thickness; failure mechanism.	5-MP, Not reported, VIC-2D & 3D [104,110]
[141]	Twenty-seven dry joint (flat, single-keyed, three-keyed) specimens of prestressed segmental bridges were subjected to push-off tests.	Joint sliding; deformation and crack analysis.	3.6-MP, Not reported, GOM 2D [109]
6. Steel Structures			
[96]	A W4 × 13 steel beam tested under three-point bending.	Beam deflection.	2-MP, Not reported, VIC-2D [104]
[95]	A wide-flange steel beam, 5 m long, 0.2 m wide, and 8 mm thick, tested under three-point bending.	Beam deflection.	8-MP, Not reported, Not reported
[16]	A five-story steel framed structure subjected to a series of dynamic experiments.	Dynamic analysis and damage detection.	2-MP, Not reported, Not reported
[50]	A hollow steel section (HSS) beam, 1.2 m long, and a 0.1 × 0.1 m section loaded under three-point bending.	Longitudinal strains; beam curvature.	18-MP, 27.8 px/mm, GeoPIV [122,123,124]
[21]	Free vibration and earthquake tests on a steel frame (0.5 length × 0.3 width × 1.1 height).	Displacement, velocity, and acceleration.	high-speed 5-MP, Not reported, Not reported
[80]	A 152 mm adhesively bonded double-cantilever beam (DCB), loaded at its free end.	Displacement and strain. displacements within a cohesive zone; rotation and shear strain.	Two 5-MP, Not reported, VIC-2D [104]
[118]	A thin 950 mm long cantilever steel beam was subjected to static loads at five locations.	Scale factor; full-field beam deflection.	1.3-MP, Not reported, Not reported
7. Slabs and Other Members			
[97]	Uniaxial tensile testing of dog-bone specimens.	Three speckle pattern configurations were used to measure the strain.	5-MP, Not reported, VIC-2D & 3D [104,110]
[142]	Three 1/3 of two-span RC bridges were tested under bidirectional earthquake conditions.	Frequency estimation, damping ratio, and mode shapes of the bridges.	5-MP, Not reported, GOM 2D [109]
[102]	Static loading of corrugated metal pipes.	Deflection and pipe profile.	18-MP, 3 px/mm–4 px/mm, GOM 2D [109]
[42,59]	Reinforced concrete RC panel subjected to diagonal tension.	Automated crack width and slip measurement.	29-MP, 2.63 px/mm, VIC-3D [110]
[101]	Bimrock disc specimens, 40 mm diameter and 15 mm thickness were tested under the tensile Brazilian disc test.	Crack initiation. Maximum shear strain; overall failure pattern of three types of bimrocks.	NA-MP, 60-fps. Not reported, Not reported
[98]	Series of semi-circular bending tests of the mine tailings rock material.	Strain distribution; mode-I fracture toughness	Not reported, 22.7 px/mm, VIC-3D [110]
[47]	Three overturned T-beam RC slabs under controlled laboratory conditions. Two of the concrete slabs (0.55 × 3.6 × 9.0 m) were stripped from an in situ full-scale Ot-slab bridge with a 9 m span and one downscaled slab.	Crack identification and assessment.	Two cameras 20-MP, 18.7-MP, 0.69 px/mm–1.72 px/mm, GOM 2D [109]
[100]	Double-L shaped RC specimens; shear push-off testing.	Crack kinematics were observed using the full-field strain.	18-MP, 24-MP, 10.8 px/mm to 11.8 px/mm, GOM 2D [109]
[143]	Six ultra-high-performance fiber-reinforced and steel rebars reinforced concrete specimens (500 mm long, 100 mm wide, and 50 mm thick) were tested using direct tensile tests.	Full-field displacement and strain; cracking behavior.	Two 9.1-MP, 13.3 px/mm, Not reported
